# Socioeconomic Position in Early Life and Childhood Cardiovascular Health Measures: An International Cross‐Cohort Study

**DOI:** 10.1161/JAHA.125.047846

**Published:** 2026-08-27

**Authors:** Kavindi Gamage, Susanna Pätsi, Tong Chen, Kate Mooney, Sylvain Sebert, Naomi Priest, Toby Mansell, David Burgner, the Barwon Infant Study Investigator Group

**Affiliations:** ^1^ The University of Melbourne Parkville VIC Australia; ^2^ Murdoch Children's Research Institute Royal Children's Hospital Parkville VIC Australia; ^3^ Research Unit of Population Health University of Oulu Linnanmaa Finland; ^4^ Department of Health Sciences University of York York UK; ^5^ Bradford Institute for Health Research, Temple Bank House, Bradford Royal Infirmary Bradford UK; ^6^ Centre for Social Research and Methods Australian National University Canberra ACT Australia

**Keywords:** ALSPAC (Avon Longitudinal Study of Parents and Children), body mass index, cardiovascular risk, children, life course, social determinants, Epidemiology, Pediatrics, Risk Factors

## Abstract

**Background:**

Cardiovascular disease is socioeconomically patterned, and risk accrues from early life. Evidence in younger populations is scarce. We investigated the extent to which early life socioeconomic position is associated with cardiovascular health in children and adolescents, and the extent of mediation by body mass index.

**Methods:**

We analyzed 5 longitudinal birth cohorts: BIS (the Barwon Infant Study; Australia; mean age 4.1 years; n=708); BiB (Born in Bradford; United Kingdom; mean age 9.3 years; n=4576); LSAC‐CP (the Longitudinal Study of Australian Children's Child Health CheckPoint; Australia; mean age 12.0 years; n=1874); NFBC1986 (the Northern Finland Birth Cohort 1986; Finland; mean age 16.0 years; n=9467); and ALSPAC (the Avon Longitudinal Study of Parents and Children; United Kingdom; mean age 17.8 years; n=4875). Exposures were neighborhood disadvantage, household socioeconomic position, and maternal education (in pregnancy/at birth). Outcomes were carotid intima‐media thickness, pulse‐wave velocity, blood pressure, and lipids (in childhood/adolescence).

**Results:**

From age 12 years, children with lower socioeconomic position had worse cardiovascular health, adjusted for age, sex, and ethnicity. For example, LSAC‐CP 12‐year‐olds in the most disadvantaged neighborhoods had higher pulse‐wave velocity (β=0.1 m/s [95% CI, 0.03–0.2 m/s]; *P*=0.003) than those in the most advantaged, and NFBC1986 16‐year‐olds whose mothers had the lowest education levels had higher triglycerides (β=0.07 mmol/L [95% CI, 0.0–0.1 mmol/L]; *P*=0.04) than those whose mothers had the highest education levels. Hypothetically intervening on body mass index would reduce 12% to 100% of some cardiovascular health differences.

**Conclusions:**

Associations between lower socioeconomic position and worse cardiovascular health are apparent from midchildhood. Prevention efforts should address intermediate mechanisms and upstream neighborhood, household, and maternal factors.

**Nonstandard Abbreviations and Acronyms**
ALSPAC: Avon Longitudinal Study of Parents and Children
BiB: Born in Bradford
BIS: Barwon Infant Study
cIMT: carotid intima‐media thickness
CVH: cardiovascular health
DBP: diastolic blood pressure
LSAC‐CP: Longitudinal Study of Australian Children's Child Health CheckPoint
NFBC1986: Northern Finland Birth Cohort 1986
PWV: pulse‐wave velocity
SBP: systolic blood pressure
SEP: socioeconomic position

**Clinical Perspective**

**What Is New?**

- This is the first and largest multicohort study to date to investigate associations between multiple indicators of socioeconomic position and cardiovascular health, across several time points in childhood and adolescence, comprehensively assessed by vascular measures and lipids.
- Associations between lower socioeconomic position and adverse cardiovascular health were observed across several high‐income contexts from 12 years of age, many of which were mediated by body mass index.

**What Are the Clinical Implications?**

- The findings of this study support clinical efforts to account for children's social context and provide equitable care to reduce the obesogenic environment and improve cardiovascular health.

Cardiovascular disease (CVD) is the leading cause of morbidity and mortality worldwide.^1^ Despite widespread secondary prevention efforts, the global burden of CVD continues to increase.^2^ In high‐income countries, there are marked population‐level socioeconomic inequalities.^1^, ^3^ Lower socioeconomic position (SEP), including lower educational attainment, lower income, and neighborhood deprivation, is associated with a higher risk of CVD, a pattern that persists across the socioeconomic gradient.^4^ As socioeconomic inequality increases globally, previous declines in CVD rates in high‐income countries are now beginning to plateau, or in some instances, reverse.^1^, ^5^

The pathogenesis of atherosclerotic CVD begins early in life. Lesions indicative of early atherosclerosis are present in utero and infancy, and are strongly associated with socioeconomically patterned maternal risk factors, such as smoking and hypercholesterolemia.^6^, ^7^ The risk of CVD accumulates across the life course, and cardiovascular health (CVH) from childhood onwards is predictive of adult CVD.^8^, ^9^ Early life is therefore increasingly recognized as a critical but largely untapped opportunity to mitigate the increasing CVD burden and associated inequalities. However, there is a paucity of data from children and adolescents. Although some studies have shown that lower SEP is associated with adverse cardiovascular phenotypes in pediatric populations,^10^, ^11^, ^12^ most include a wide range of ages in the analytical sample and focus on singular exposures and outcomes. Disaggregating age groups and considering multiple dimensions of the socioeconomic environment may improve understandings of mechanistic pathways and inform timely interventions.

In this study, we investigated the extent to which neighborhood disadvantage, household SEP, and maternal education in early life are associated with several vascular and lipid measures of CVH across childhood and adolescence. As overweight and obesity contribute to CVD risk,^13^ we also investigated the extent to which body mass index (BMI) explains these associations, and thus, its utility as a potential intervention target. We present data from 5 population‐derived birth cohort studies from Australia, Finland, and the United Kingdom, with participants aged 4 to 18 years.

## METHODS

### Study Cohorts

We used data from 5 population‐derived birth cohorts: BIS (the Barwon Infant Study; Australia; mean age at outcome measurement, 4.1 years; n=708); BiB (Born in Bradford; United Kingdom; mean age, 9.3 years; n=4576); LSAC‐CP (the Longitudinal Study of Australian Children's Child Health CheckPoint; Australia; mean age, 12.0 years; n=1874); NFBC1986 (the Northern Finland Birth Cohort 1986; Finland; mean age, 16.0 years; n=9467); and ALSPAC (the Avon Longitudinal Study of Parents and Children; United Kingdom; mean age, 17.8 years; n=4875). Features of study design, geographic setting, participants, and recruitment have been previously described and are presented in Data S1.^14^, ^15^, ^16^, ^17^, ^18^ We included all participants recruited at baseline and who attended a study visit at the selected wave of data collection. BIS data are available under restricted access on scientific and ethical grounds by the BIS Steering Committee and provided through a collaborative research agreement. A description of the available data is available on the cohort website: https://barwoninfantstudy.org.au. BiB data are available under restricted access through application on the study website, where information on the available data is also available: https://borninbradford.nhs.uk/research/how‐to‐access‐data/. Data from LSAC‐CP are available under restricted access and may be obtained by request through the Australian Data Archive (https://ada.edu.au/) for researchers in Australia affiliated with an approved research body. NFBC1986 data are available under restricted access through application on the study website (https://www.oulu.fi/en/university/faculties‐and‐units/faculty‐medicine/northern‐finland‐birth‐cohorts‐and‐arctic‐biobank/nfbc‐aineistopyynto) and provided through a collaborative research agreement with the University of Oulu. The informed consent obtained from ALSPAC participants does not allow the data to be made available through any third party–maintained public repository. Supporting data are available from ALSPAC on request under the approved proposal number, B4806. Full instructions for applying for data access can be found here: http://www.bristol.ac.uk/alspac/researchers/access/. The ALSPAC website contains details of all available data, including a data dictionary and variable search tool (http://www.bristol.ac.uk/alspac/researchers/our‐data/).

### Measurement of Early‐Life SEP

We included 3 indicators of SEP, measured at baseline (pregnancy or infancy) in all cohorts except ALSPAC (in which neighborhood disadvantage was measured at 7–9 years): neighborhood disadvantage, household SEP, and maternal education. We harmonized measures across cohorts such that each indicator had the same number of categories. Data sources and cutoffs were cohort specific.

We measured neighborhood disadvantage, where available, using preexisting indexes that rank areas based on the socioeconomic characteristics of inhabitants. In BIS and LSAC‐CP, this was the Socio‐Economic Indexes for Areas Index of Relative Socio‐Economic Advantage and Disadvantage.^19^ In BiB and ALSPAC, this was the English Index of Multiple Deprivation.^20^ In NFBC1986, we derived an index using Paavo postal code area data collected by Statistics Finland.^21^ Cohort‐specific tertiles were derived for each index.

We operationalized household SEP as a binary measure using absolute values of household income or reports of the family financial situation obtained from questionnaire data. In BIS and LSAC‐CP, we used household income, with cutoffs at the value closest to the population median household income at the time of measurement. In BiB, mothers reported on how the family was doing financially. We dichotomized households as “financially secure” if mothers responded that they were “living comfortably” or “doing alright,” or “financially struggling” if mothers responded that they were “just about getting by,” “finding it quite difficult,” or “finding it very difficult.” We also derived a 4‐category sensitivity measure according to previous work,^22^ in which the final 2 responses were collapsed into a single “financially insecure” category. In ALSPAC, mothers reported on their ability to afford accommodation, food, and heating. We categorized households as experiencing “financial difficulties” if there was difficulty affording any of these items, and “no financial difficulties” if not. In NFBC1986, we used parental occupational class as a proxy and dichotomized the variable as “high” or “low.”

We measured maternal education as the highest qualification obtained. We derived 4 categories for each cohort: in BIS and LSAC‐CP, less than year 12, trade or diploma, bachelor's degree, and postgraduate degree; in BiB, <5 general certificates of secondary education or equivalent, 5 general certificates of secondary education or equivalent, A levels or other certification, or a university degree; in NFBC1986, <10 years of school, any vocational school or college, matriculation, or a university degree; and in ALSPAC, Certificate of Secondary Education or vocation, O levels, A levels, or university degree.

Further details on the measurement and operationalization of these indicators are presented in Data S2.

### Measurement of CVH Measures

We selected vascular and lipid CVH measures from 1 time point in childhood or adolescence in each cohort to obtain a range of age groups. In BIS, this was at a mean age of 4.1 years; in BiB, 9.3 years; in LSAC‐CP, 12.0 years; in NFBC1986, 16.0 years; and in ALSPAC, 17.8 years. Vascular measures, where available, included maximum carotid intima‐media thickness (cIMT; measured in mm); aortic‐ or carotid‐femoral pulse‐wave velocity (PWV; m/s); systolic blood pressure (SBP; mm Hg); and diastolic blood pressure (DBP; mm Hg). Lipid measures included low‐density lipoprotein cholesterol (measured in mmol/L); high‐density lipoprotein cholesterol (HDL‐C; mmol/L); triglycerides (mmol/L); non–HDL‐C (mmol/L); and remnant cholesterol (mmol/L). Details on the collection of these measures are available in Data S3.

### Measurement of BMI

At each time point, study investigators measured BMI using the standard calculation of weight (in kilograms) divided by height (in meters) squared. Investigators measured weight and height either 1 or 2 times (and the average was taken), as previously reported.^23^, ^24^, ^25^, ^26^, ^27^

### Measurement of Covariates and Confounders

Covariates were age at outcome measurement (in years) and sex assigned at birth (male or female). Ethnicity was an additional baseline confounder. As recommended, we define ethnicity as a social construct with no biological basis.^28^, ^29^ In BIS and LSAC‐CP, we classified participants as Anglo‐European or minoritized ethnic group. In BiB, we classified participants as Pakistani, White‐British, or other (if otherwise classified). In ALSPAC, we classified participants as White or non‐White. In NFBC1986, information on ethnicity was not collected. Details on the measurement of intermediate confounders (included in the mediation models) are available in Data S4.

### Statistical Analysis

We conducted data analysis from May 2024 to June 2025. We analyzed each cohort separately, as we were interested in the timing of associations by age group. We summarized participant characteristics. Before analysis, we conducted multiple imputation by chained equations to address missingness in all study variables (Table). Further detail is presented in Data S5. The primary analysis used imputed data; we obtained estimates using complete‐case data as a sensitivity analysis. To hypothesize the causal pathways between exposures, the mediator, outcomes, covariates, and confounders, we developed a directed acyclic graph (Figure S1).

**Table 1:** Cohort Characteristics

Characteristic	BIS (N=708)		BiB (N=4576)		LSAC‐CP (N=1874)		NFBC1986 (N=9467)		ALSPAC (N=4875)	
	Value	No.	Value	No.	Value	No.	Value	No.	Value	No.
Age at outcome assessment, y	4.1±0.2	708	9.3±1.0	3264	12.0±0.4	1874	16.0±0.5	6631	17.8±0.4	4875
Sex										
Female	332 (46.9)	708	2228 (48.7)	4576	919 (49.0)	1874	4583 (48.4)	9466	2743 (56.3)	4875
Male	376 (53.1)		2348 (51.3)		955 (51.0)		4883 (51.6)		2132 (43.7)	
Ethnicity*										
White or Anglo‐European ethnic group	639 (94.8)	674			1543 (89.9)	1717			4417 (95.6)	4621
Minoritized ethnic group	35 (5.2)				174 (10.1)				204 (4.4)	
White‐British			1330 (29.0)	3826						
Pakistani origin			2496 (54.5)							
Maternal educational attainment†										
High	145 (20.6)	705	1241 (27.8)	4468	182 (9.6)	1893	518 (6.3)	8195	870 (18.5)	4694
Medium	267 (37.9)		908 (20.3)		496 (26.2)		1835 (22.3)		1307 (27.8)	
Medium‐low	172 (24.4)		1327 (29.7)		764 (40.4)		3662 (44.7)		1608 (34.2)	
Low	121 (17.2)		992 (22.2)		431 (22.8)		2180 (26.6)		909 (19.4)	
Neighborhood disadvantage‡										
Low disadvantage	246 (35.1)	701	1543 (33.7)	4572	570 (30.4)	1874	3014 (33.3)	9040	1323 (36.2)	3656
Medium disadvantage	243 (34.7)		1521 (33.3)		664 (35.4)		3013 (33.3)		1353 (37.0)	
Highest disadvantage	212 (30.2)		1508 (33.0)		640 (34.2)		3013 (33.3)		980 (26.8)	
Household socioeconomic position§										
High	512 (73.7)	694	3127 (68.7)	4550	620 (34.5)	1796	8102 (85.6)	9467	3019 (66.0)	4572
Low	182 (26.2)		1423 (31.3)		1176 (65.5)		1365 (14.4)		1553 (34.0)	
Body mass index, kg/m^2^	15.6±2.1	707	17.6±3.5	3225	19.2±3.4	1871	21.2±3.5	6767	22.8±4.2	4733
Carotid intima‐media thickness, mm||	0.6±0.05	619	NA		0.6±0.05	1485	NA		0.4±0.05	4358
Pulse‐wave velocity, m/s	4.0±0.4	588	NA		4.5±0.6	1803	NA		5.8±0.7	3664
Systolic blood pressure, mm Hg	107.1±8.7	558	103.7±12.5	3910	108.1±8.0	1777	115.5±12.8	6836	118.5±10.8	4378
Diastolic blood pressure, mm Hg	64.6±6.8	558	65.1±11.6	3910	62.4±5.8	1777	67.8±7.7	6777	63.9	4378
Low‐density lipoprotein cholesterol, mmol/L	0.9±0.3	509	1.4±0.3	1759	1.4±0.3	1179	1.5±0.5	5470	1.0±0.4	2987
High‐density lipoprotein cholesterol, mmol/L	1.5±0.2	509	1.4±0.3	1759	1.4±0.3	1179	1.5±0.3	5470	1.4±0.2	2987
Total triglycerides, mmol/L	0.9±0.3	509	1.1±0.5	1759	1.2±0.6	1179	1.0±0.4	5470	0.9±0.3	2987
Non–high‐density lipoprotein cholesterol, mmol/L	1.6±0.5	509	2.6±0.6	1759	2.7±0.6	1179	3.0±0.8	5470	2.1±0.6	2987
Remnant cholesterol, mmol/L	0.7±0.2	509	1.2±0.3	1759	1.3±0.4	1179	1.5±0.4	5470	1.1±0.3	2987

Continuous, normally distributed variables are reported as mean±SD, and categorical variables are reported as number (percentage). ALSPAC indicates Avon Longitudinal Study of Parents and Children; BiB, Born in Bradford; BIS, Barwon Infant Study; LSCAC‐CP, Longitudinal Study of Australian Children's Child Health CheckPoint; NA, not available; and NFBC1986, Northern Finnish Birth Cohort 1986.

* Majority ethnic group in BIS and LSAC‐CP defined as Anglo/European; and in ALSPAC, White. Minoritized ethnic group in BIS and LSAC‐CP defined as other parental origins; and in ALSPAC, non‐White.

^†^ In BIS and LSAC‐CP, high indicates a postgraduate degree; medium, a bachelor's degree; medium‐low, a trade, certificate, or diploma; and low, year 12 or less. In BiB, high indicates a university degree; medium, A levels or equivalent; medium‐low, 5 general certificates of secondary education (GCSEs) or equivalent; and low, <5 GCSEs or equivalent. In NFBC1986, high indicates a university degree; medium, matriculation; medium‐low, any vocational school or college; and low, <10 years of schooling. In ALSPAC, high indicates a university degree; medium, A levels; medium‐low, O levels; and low, certificate of secondary education or vocation.

^‡^ In BIS and LSAC‐CP, neighborhood disadvantage was measured using the Socioeconomic Indexes for Areas (Australia); in BiB and ALSPAC, using the Index of Multiple Deprivation (England); and in NFBC1986, Paavo data (Finland).

^§^ In BIS and LSAC‐CP, household socioeconomic position was measured using household income (high vs low); in BiB, financial security (secure vs financially struggling); in NFBC1986, parental occupational class (high vs low); and in ALSPAC, financial difficulties (any vs none).

^||^ All measurements are of mean maximum carotid intima‐media thickness, except in ALSPAC, which used mean carotid intima‐media thickness.

We estimated associations between each SEP indicator in early life and each CVH measure in childhood/adolescence using multivariable linear regression. We set the most socioeconomically advantaged category for each indicator as the reference group, and included unadjusted and adjusted (for age, sex, and ethnicity) models. We did not adjust for SEP at outcome measurement as it is likely to be a mediator on the causal pathway between early‐life SEP and later CVH. We present estimates with 95% CIs. In secondary analyses, we included a sex‐SEP interaction term in models to examine effect modification by sex and conducted sex‐stratified analysis to characterize any differences. We considered sex to encompass both biologically sex‐linked and socially gendered mechanisms.^30^ We could not examine the effects of gender alone as it was not consistently measured in all the cohorts. We investigated effect modification by ethnicity in BiB to estimate any differential SEP relationships in Pakistani and White‐British children. In BiB, we additionally conducted sensitivity analysis using a 4‐category financial security measure, as previously used.^22^

We used mediation analysis using an interventional effects approach (defined by mapping to a hypothetical randomized trial)^31^ to evaluate the mediating role of BMI and the efficacy of hypothetical interventions. This method is based on a Monte Carlo simulation‐based g‐computation approach and can also account for exposure–induced mediator–outcome (intermediate) confounding (Figure S1).^32^, ^33^ We estimated the total effect and the interventional indirect effect through BMI. This indirect effect could be considered the extent to which a hypothetical intervention that shifts the distribution of BMI from that in a lower (exposed) SEP group to be the same as that in the reference (unexposed) SEP group would reduce differences in the CVH measures. We present these relative differences as percentages (the proportion of the total causal effect that is the interventional indirect effect). Further detail on the mediation analysis is available in Data S5.

We performed all statistical analyses in R.4.1.2.^34^ This study was approved by The Royal Children's Hospital Melbourne Human Research Ethics Committee.

## RESULTS

This study included a total of 21 500 participants aged 4 to 18 years: in BIS, 708 children (mean±SD age, 4.1±0.2 years; 332 girls [46.9%]); in BiB, 4576 children (mean±SD age, 9.3±1.0 years; 2228 girls [48.7%]); in LSAC‐CP, 1874 children (mean±SD age, 12.0±0.4 years; 919 girls [49.0%]); in NFBC1986, 9467 adolescents (mean±SD age, 16.0±0.5 years; 4583 girls [48.4%]); and in ALSPAC, 4875 adolescents (mean±SD age, 17.8±0.4 years; 2743 girls [56.3%]) (Table).

### Neighborhood Disadvantage and CVH

Associations between neighborhood disadvantage and child and adolescent CVH were mixed across cohorts. Greater neighborhood disadvantage was linked to worse CVH in LSAC‐CP, NFBC1986, and ALSPAC, but these CVH measures differed between cohorts.

In BIS, associations were in the inverse direction: BIS children in medium disadvantage neighborhoods had lower SBP (β=−2.0 mm Hg [95% CI, −3.8 to −0.3 mm Hg]; *P=*0.02) and DBP (β=−1.7 mm Hg [95% CI, −3.1 to −0.3 mm Hg]; *P=*0.01) than those in the most advantaged neighborhoods (Figure 1A). In BiB, estimates were imprecise, and CIs included both meaningful benefit and harm (Figure 1B).

**Figure 1:**
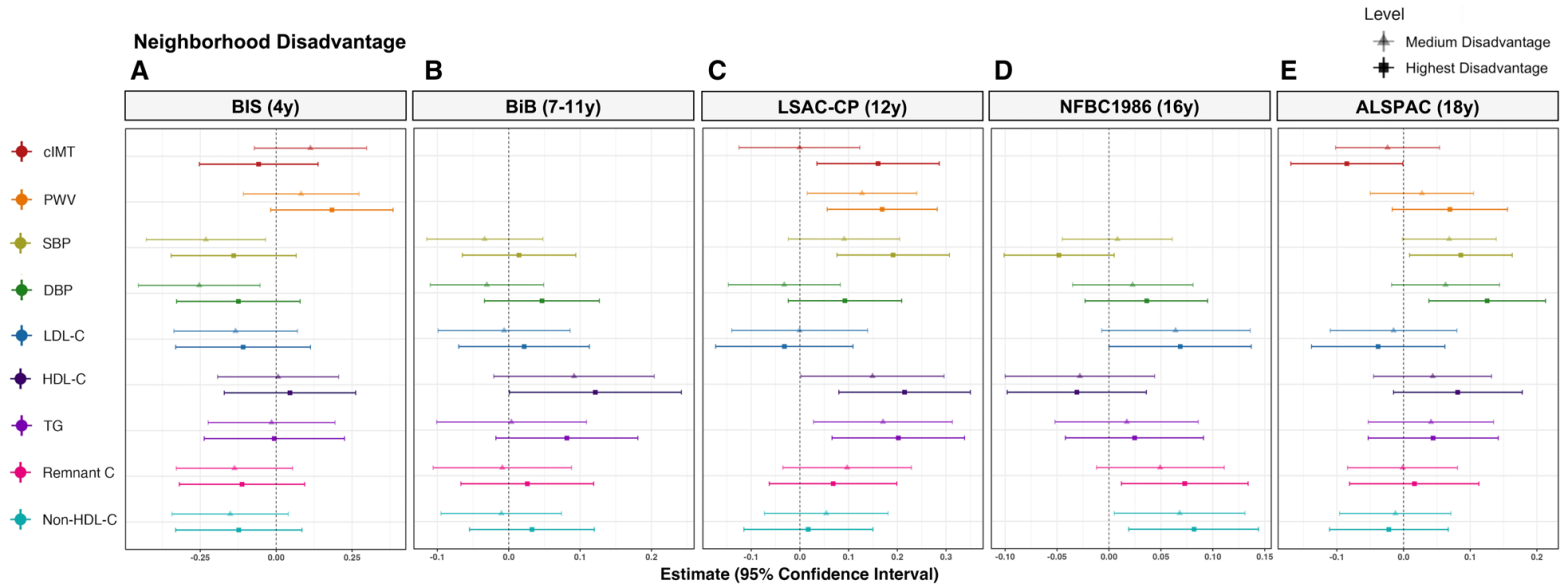
Associations between neighborhood disadvantage in early life (pregnancy or infancy) and CVH measures in childhood and adolescence. **A**, Results for BIS at 4 years. **B**, Results for BiB at 7 to 11 years. **C**, Results for LSAC‐CP at 12 years. **D**, Results for NFBC1986 at 16 years. **E**, Results for ALSPAC at 18 years. Estimates (triangles and squares) are the mean difference in the outcome measure in SD units compared with the reference group (low neighborhood disadvantage, indicated by the dotted vertical line), adjusted for age, sex, and ethnicity. Horizontal bars indicate 95% CIs of estimates. HDL‐C has been reverse coded. ALSPAC indicates the Avon Longitudinal Study of Parents and Children; BiB, Born in Bradford; BIS, Barwon Infant Study; cIMT, carotid intima‐media thickness; CVH, cardiovascular health; DBP, diastolic blood pressure; HDL‐C, high‐density lipoprotein cholesterol; LDL‐C, low‐density lipoprotein cholesterol; LSAC‐CP, Longitudinal Study of Australian Children's Child Health CheckPoint; NFBC1986, Northern Finland Birth Cohort 1986; PWV, pulse‐wave velocity; Remnant C, remnant cholesterol; SBP, systolic blood pressure; and TG, total triglycerides.

In LSAC‐CP, children in the most disadvantaged neighborhoods had higher cIMT (β=0.008 mm [95% CI, 0.002–0.013 mm]; *P*=0.01), PWV (β=0.10 m/s [95% CI, 0.03–0.16 m/s]; *P*=0.003), SBP (β=1.5 mm Hg [95% CI, 0.6–2.4 mm Hg]; *P*=0.001), and triglycerides (β=0.1 mmol/L [95% CI, 0.1–0.2 mmol/L]; *P=*0.004), and lower HDL‐C (β=−0.06 mmol/L [95% CI, −0.10 to −0.02 mmol/L]; *P=*0.002), than those in the most advantaged neighborhoods (Figure 1C). Children in medium disadvantage neighborhoods also had higher PWV (β=0.1 m/s [95% CI, 0.0–0.1 m/s]; *P=*0.03) and triglycerides (β=0.1 mmol/L [95% CI, 0.02–0.2 mmol/L]; *P=*0.02) than those in the most advantaged neighborhoods. In NFBC1986, adolescents in the most disadvantaged neighborhoods had higher non–HDL‐C (β=0.07 mmol/L [95% CI, 0.02–0.12 mmol/L]; *P=*0.01) and remnant cholesterol (β=0.027 mmol/L [95% CI, 0.005–0.049 mmol/L]; *P*=0.02) than those in the most advantaged neighborhoods (Figure 1D). Adolescents in medium disadvantaged neighborhoods also had higher non–HDL‐C (β=0.1 mmol/L [95% CI, 0.0–0.1 mmol/L]; *P=*0.04) than those in the most advantaged neighborhoods. In ALSPAC, adolescents in the most disadvantaged neighborhoods had higher SBP (β=0.9 mm Hg [95% CI, 0.1–1.8 mm Hg]; *P*=0.03) and DBP (β=0.8 mm Hg [95% CI, 0.2–1.4 mm Hg]; *P*=0.005) than those in the most advantaged neighborhoods (Figure 1E).

Subgroup analysis showed that girls in the most disadvantaged neighborhoods in NFBC1986 may have higher triglycerides (β=0.1 mmol/L [95% CI, −0.0 to 0.1 mmol/L]; *P=*0.07), but not boys (β=0.02 mmol/L [95% CI, −0.05 to 0.07 mmol/L]; *P=*0.40) (difference: β=−0.1 mmol/L [95% CI, −0.1 to 0.0 mmol/L]; *P=*0.06) (Tables S1–S3).

Mediation analysis indicated that BMI explained some of the relationship between neighborhood disadvantage and CVH in the older cohorts. In LSAC‐CP, a (hypothetical) intervention to shift the distribution of BMI in children from the most disadvantaged neighborhoods to the levels of those from the least disadvantaged neighborhoods would lead to a 20% reduction in PWV differences (interventional indirect effect [IIE]=0.02 m/s [95% CI, 0.0–0.04 m/s]; *P=*0.008), a 53% reduction in SBP differences (IIE=0.8 mm Hg [95% CI, 0.4–1.2 mm Hg]; *P=*<0.001), and a 15% reduction in HDL‐C differences (IIE=−0.009 mmol/L [95% CI, −0.02 to 0.0 mmol/L]; *P=*0.03) (Tables S4 and S5). Shifting the distribution of BMI in children from medium disadvantage neighborhoods to the levels of those from the most advantaged neighborhoods would also lead to a 43% reduction in PWV differences (IIE=0.03 m/s [95% CI, 0.006–0.05 m/s]; *P=*0.01) (Table S4). In ALSPAC, shifting the distribution of BMI in adolescents from the most disadvantaged neighborhoods to the levels of those in the least disadvantaged neighborhoods would lead to a complete reduction in SBP differences (IIE=1.0 mm Hg [95% CI, 0.6–1.3 mm Hg]; *P=*<0.001) and a 75% reduction in DBP differences (IIE=0.6 mm Hg [95% CI, 0.4–0.8 mm Hg]; *P*=0.005) (Table S6). CIs were wide in BIS, BiB, and NFBC1986 (Tables S7–S12).

### Household SEP and CVH

Lower household SEP was not associated with CVH in BIS apart from in 1 sex stratified model. In BiB, an inverse association was observed: children in families struggling financially had lower DBP (β=**−**0.8 mm Hg [95% CI, −1.6 to −0.1 mm Hg]; *P*=0.03) (Figure 2B). In LSAC‐CP, NFBC1986, and ALSPAC, lower household SEP was associated with higher SBP, and in some cases, certain lipid measures. For example, LSAC‐CP children in lower‐income households had higher SBP (β=0.9 mm Hg [95% CI, 0.1–1.7 mm Hg]; *P=*0.02), lower HDL‐C (β=−0.04 mmol/L [95% CI, −0.08 to −0.01 mmol/L]; *P*=0.01), and higher triglycerides (β=0.1 mmol/L [95% CI, 0.0–0.2 mmol/L]; *P*=0.03) compared with their more advantaged counterparts (Figure 2C). Similarly, NFBC1986 adolescents with parents with lower parental occupational class had higher SBP (β=2.0 mm Hg [95% CI, 1.2–2.8 mm Hg]; *P=*<0.001), DBP (β=0.8 mm Hg [95% CI, 0.2–1.3 mm Hg]; *P*=0.005), and non–HDL‐C (β=0.1 mmol/L [95% CI, 0.0–0.1 mmol/L]; *P*=0.03) (Figure 2D). ALSPAC adolescents in households experiencing financial difficulties also had higher SBP (β=0.7 mm Hg [95% CI, 0.1–1.3 mm Hg]; *P=*0.02) (Figure 2E).

**Figure 2:**
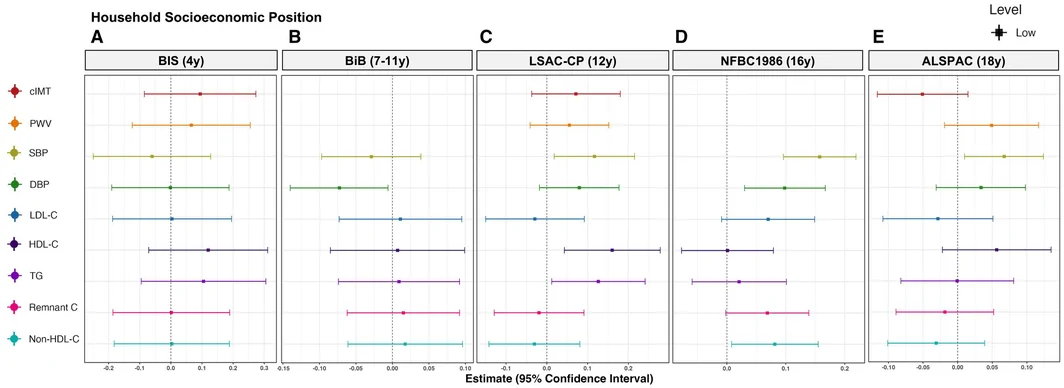
Associations between household SEP in early life (pregnancy or infancy) and CVH measures in childhood and adolescence. **A**, Results for BIS at 4 years. **B**, Results for BiB at 7 to 11 years. **C**, Results for LSAC‐CP at 12 years. **D**, Results for NFBC1986 at 16 years. **E**, Results for ALSPAC at 18 years. Squares are the point estimates of the mean difference in the outcome measure in SD units in low household SEP groups compared with the reference group (high household SEP), indicated by the dotted vertical line, adjusted for age, sex, and ethnicity. Household SEP was measured in each cohort as follows: BIS, household income (reference group: higher household income); BiB, financial security (reference group: financially secure); LSAC‐CP, household income (reference group: higher household income); NFBC1986, parental occupational class (reference group: high parental occupational class); and ALSPAC, financial difficulties (reference group: no financial difficulties). Horizontal bars indicate 95% CIs of estimates. HDL‐C has been reverse coded. ALSPAC indicates Avon Longitudinal Study of Parents and Children; BiB, Born in Bradford; BIS, Barwon Infant Study; cIMT, carotid intima‐media thickness; CVH, cardiovascular health; DBP, diastolic blood pressure; HDL‐C, high‐density lipoprotein cholesterol; LDL‐C, low‐density lipoprotein cholesterol; LSAC‐CP, Longitudinal Study of Australian Children's Child Health CheckPoint; NFBC1986, Northern Finland Birth Cohort 1986; PWV, pulse‐wave velocity; Remnant C, remnant cholesterol; SBP, systolic blood pressure; SEP, socioeconomic position; and TG, total triglycerides.

In sex‐stratified models in BIS, boys in lower‐income households had higher cIMT (β=0.014 mm [95% CI, 0.002–0.027 mm]; *P*=0.03), but not girls (β=−0.01 mm [95% CI, −0.02 to 0.01 mm]; *P*=0.31), with a difference (β=0.019 mm [95% CI, 0.003–0.036 mm]; *P*=0.02) (Tables S13–S15). Sensitivity analysis in BiB showed that specifically children in families “just about getting by” had lower DBP than those “living comfortably” (β=−1.0 mm Hg [95% CI, −2.0 to 0.0 mm Hg]; *P*=0.05) (Table S16).

There was some evidence of mediation by BMI. A (hypothetical) intervention in LSAC‐CP to shift the distribution of BMI in lower‐income household children to be the same as those in higher‐income households would reduce 78% of SBP differences (IIE=0.7 mm Hg [95% CI, 0.3–1.0 mm Hg]; *P*=<0.001), 23% of HDL‐C differences (IIE=−0.009 mmol/L [95% CI, −0.020 to −0.002 mmol/L]; *P*=0.02), and 22% of triglyceride differences (IIE=0.02 mmol/L [95% CI, 0.002–0.04 mmol/L]; *P=*0.03) (Tables S4 and S5). Shifting the distribution of BMI in ALSPAC adolescents in families experiencing financial difficulties to be the same as those with none would reduce 57% of SBP differences (IIE=0.4 mm Hg [95% CI, 0.2–0.7 mm Hg]; *P*=<0.001) (Table S6). There was no evidence of mediation in BIS, BiB, and NFBC1986, as CIs were wide (Tables S7–S12).

### Maternal Education and CVH

Associations between maternal education and CVH across the cohorts were also mixed. In the main models, there was no evidence of any associations in BIS and BiB. In LSAC‐CP, NFBC1986, and ALSPAC, lower maternal education was associated with worse CVH, although measures differed between cohorts.

LSAC‐CP children whose mothers had less than a postgraduate degree had higher SBP (less than year 12: β=2.0 mm Hg [95% CI, 0.6–3.4 mm Hg]; *P=*<0.001; trade/diploma/certificate: β=2.4 mm Hg [95% CI, 1.1–3.7 mm Hg]; *P=*0.004; bachelor's degree: β=1.4 mm Hg [95% CI, 0.0–2.8 mm Hg]; *P=*0.05) and DBP (less than year 12: β=1.4 mm Hg [95% CI, 0.3–2.4 mm Hg]; *P=*0.009; trade/diploma/certificate: β=1.6 mm Hg [95% CI, 0.7–2.6 mm Hg]; *P*=0.005; bachelor's degree: β=1.4 mm Hg [95% CI, 0.4–2.4 mm Hg]; *P=*0.005) (Figure 3C). In NFBC1986, adolescents with mothers with less than 10 years of schooling had higher triglycerides than those whose mothers had a university degree (β=0.07 mmol/L [95% CI, 0.0–0.1 mmol/L]; *P*=0.04) (Figure 3D). Finally, in ALSPAC, adolescents with mothers with a vocational qualification/certificate of secondary education had higher PWV (β=0.08 m/s [95% CI, 0.01–0.2 m/s]; *P*=0.02), SBP (β=2.3 mm Hg [95% CI, 1.4–3.3 mm Hg]; *P=<*0.001), and DBP (β=1.7 mm Hg [95% CI, 1.1–2.3 mm Hg]; *P*<0.001) than those whose mothers had a university degree. Adolescents whose mothers had completed O levels also had higher SBP (β=1.2 mm Hg [95% CI, 0.4–2.1 mm Hg]; *P=*0.004) and DBP (β=1.0 mm Hg [95% CI, 0.4–1.5 mm Hg]; *P=*0.001). The results also showed that ALSPAC adolescents whose mothers had a vocational qualification/certificate of secondary education had lower cIMT (β=−0.008 mm [95% CI, −0.013 to −0.002 mm]; *P=*0.004) (Figure 3E).

**Figure 3:**
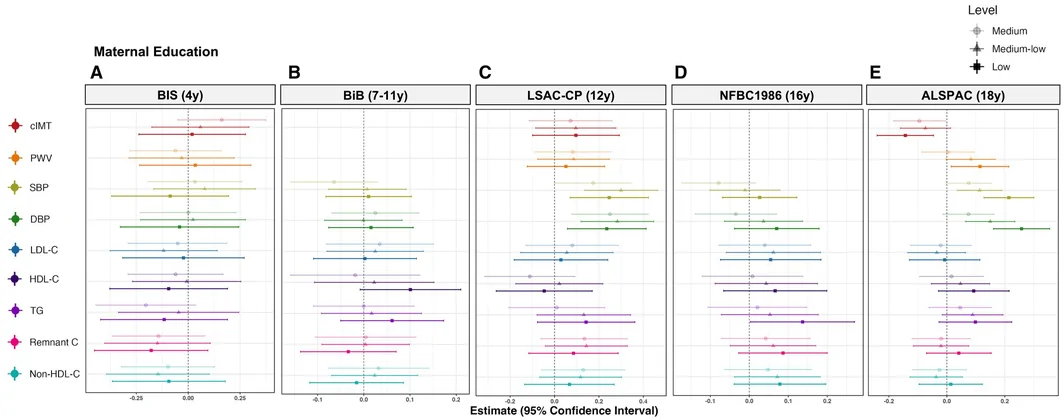
Associations between maternal education in early life (pregnancy or infancy) and CVH measures in childhood and adolescence. **A**, Results for BIS at 4 years. **B**, Results for BiB at 7 to 11 years. **C**, Results for LSAC‐CP at 12 years. **D**, Results for NFBC1986 at 16 years. **E**, Results for ALSPAC at 18 years. Estimates (circles, triangles, and squares) are the mean difference in the outcome measure in SD units in lower maternal education groups compared with the reference group, indicated by the dotted vertical line, adjusted for age, sex, and ethnicity. In BIS, the reference group was a postgraduate degree; medium, a bachelor's degree; medium‐low, a trade, certificate, or diploma; and low, year 12 or less. In BiB, the reference group was a university degree; medium, A levels or equivalent; medium‐low, 5 GCSEs or equivalent; and low, <5 GCSEs or equivalent. In LSAC‐CP, the reference group was a postgraduate degree; medium, a bachelor's degree; medium‐low, a trade, certificate, or diploma; and low, year 12 or less. In NFBC1986, the reference group was a university degree; medium, matriculation; medium‐low, any vocational school or college; and low, <10 years of schooling. In ALSPAC, the reference group was a university degree; medium, A levels; medium‐low, O levels; and low, CSE or vocation. Horizontal bars indicate 95% CIs of estimates. HDL‐C has been reverse coded. ALSPAC indicates Avon Longitudinal Study of Parents and Children; BiB, Born in Bradford; BIS, Barwon Infant Study; cIMT, carotid intima‐media thickness; CSE, certificate of secondary education; CVH, cardiovascular health; DBP, diastolic blood pressure; GCSE, general CSE; HDL‐C, high‐density lipoprotein cholesterol; LDL‐C, low‐density lipoprotein cholesterol; LSAC‐CP, Longitudinal Study of Australian Children's Child Health CheckPoint; NFBC1986, Northern Finland Birth Cohort 1986; PWV, pulse‐wave velocity; Remnant C, remnant cholesterol; SBP, systolic blood pressure; and TG, total triglycerides.

In BIS, subgroup analysis indicated associations in the opposite direction: girls whose mothers had completed year 12 or less had lower remnant cholesterol (β=2.0 mmol/L [95% CI, 0.6–3.4 mmol/L]; *P=*0.01) and lower non−HDL‐C (β=2.0 mmol/L [95% CI, 0.6–3.4 mmol/L]; *P=*0.01); difference with boys was 0.1 mmol/L (95% CI, 0.0–0.2 mmol/L; *P=*0.10) and 0.3 mmol/L (95% CI, 0.0–0.6 mmol/L; *P=*0.04), respectively (Tables S13–S15). In ALSPAC adolescents, the above association between maternal education and cIMT was specific to girls (β=−0.011 mm [95% CI, −0.018 to −0.005 mm]; *P*=0.003; difference with boys: β=0.009 mm [95% CI, −0.002 to 0.019 mm]; *P*=0.09) (Tables S17–S19). The association between having a mother with a vocational/certificate of secondary education–level education and SBP and DBP was also stronger for girls (eg, DBP in girls: β=2.3 mm Hg [95% CI, 1.4–3.2 mm Hg]; *P*=<0.001; DBP in boys: β=0.9 mm Hg [95% CI, −0.1 to 1.8 mm Hg]; *P*=0.07; difference: β=−1.4 m/s [95% CI, −2.6 to −0.1 m/s]; *P*=0.03) (Tables S17–S19).

There was evidence for mediation in part by BMI for some of these relationships. In LSAC‐CP, a (hypothetical) intervention to shift the distribution of BMI in children with mothers who had completed year 12 or less to be the same as those whose mothers had completed a postgraduate degree would reduce 42% of SBP differences (IIE=0.8 mm Hg [95% CI, 0.2–1.4 mm Hg]; *P*=0.01) and 21% of DBP differences (IIE=0.3 mm Hg [95% CI, 0.0–0.6 mm Hg]; *P*=0.04). Similarly, shifting the distribution of BMI in children whose mothers had a trade/diploma/certificate to be the same as in those whose mothers had a postgraduate degree would reduce 42% of SBP differences (IIE=1.0 mm Hg [95% CI, 0.4–1.5 mm Hg]; *P*=0.001) and 12% of DBP (IIE=0.2 mm Hg [95% CI, 0.0–0.4 mm Hg]; *P*=0.02) (Table S4). Shifting the distribution of BMI in ALSPAC adolescents with mothers with a vocation/certificate of secondary education to be the same as those with mothers with a university degree would reduce SBP differences by 48% (IIE=1.1 mm Hg [95% CI, 0.8–1.5 mm Hg]; *P*=<0.001) and DBP differences by 41% (IIE=0.7 mm Hg [95% CI, 0.5–1.0 mm Hg]; *P*=<0.001). There was a similar if not more marked result of shifting the distribution of BMI in adolescents with mothers with O levels to be the same as those with mothers with a university degree: SBP differences would be reduced by 100% (IIE=0.8 mm Hg [95% CI, 0.5–1.1 mm Hg]; *P*=<0.001) and DBP differences by 50% (IIE=0.5 mm Hg [95% CI, 0.3–0.7 mm Hg]; *P*=<0.001) (Table S6). CIs were wide in BIS, BiB, and NFBC1986 (Tables S7–S12).

Estimates using complete case data were comparable to those from the primary analyses (Tables S20–S24). In ethnicity‐stratified analyses in BiB, CIs were wide, and there was little evidence of a difference in associations between SEP indicators and CVH in Pakistani versus White‐British children (Tables S25–S27). Estimates for the primary results presented in Figures 1, 2, and 3 are also available in table format in Tables S28, S29, S30, S31, and S32.

## DISCUSSION

This multicohort study of 21 500 participants, aged 4 to 18 years, from 5 longitudinal birth cohorts suggests that lower early‐life SEP may be linked to worse CVH from midchildhood. These associations were observed in different population contexts from Australia, Finland, and the United Kingdom, and for a range of vascular and lipid measures. We observed associations for SEP at the neighborhood, household and individual level. Mediation analysis indicated that between 15% and 100% of relative differences in CVH measures could be reduced by a hypothetical intervention on BMI. However, associations between specific exposures and outcomes were not consistent across the cohorts, potentially attributable to age, period, and cohort effects, which we explore further here.

We report the largest study to investigate these questions to date, in which we disaggregated age groups to estimate associations between individual, household, and neighborhood‐level socioeconomic indicators and multiple measures of CVH across several population‐based cohorts. Our findings align with previous studies, which have shown that lower SEP is associated with higher cIMT,^35^ higher blood pressure (BP),^11^, ^36^, ^37^ and an adverse lipid profile^38^, ^39^ in children and adolescents. We provide further evidence that socioeconomic inequalities in the risk and pathogenesis of CVD may originate early in life, well before the presentation of clinical disease.

In this study, we did not use clinical cutoffs for the outcome measures, which currently only exist for BP and lipid levels in pediatric populations.^40^, ^41^ Rather than identify which children were at risk, we aimed to explore the social production of inequalities in early CVH and quantify differences between socially stratified groups. This is particularly important given recent evidence indicating that higher CVD risk in childhood is associated with future CVD events, independent of adult CVD risk, per unit increase in mean risk score.^8^ When clinically informed risk factor categories were used, there was a higher risk of adult CVD for not only the high‐risk but high‐normal and high‐acceptable groups. The differences observed in our study, although small, may thus still contribute to population‐level inequalities in clinical CVD later in life, even if socioeconomic conditions improve into adulthood.^42^ This is supported by the large body of evidence on the importance of maintaining optimal CVH throughout the life course.^43^, ^44^ Therefore, even small reductions in inequalities in CVH in childhood and adolescence may accumulate to produce meaningful population‐level reductions in inequalities in CVD.

In this study, associations were observed in opposite directions in BIS and BiB (ages 4–11 years). It is difficult to ascertain whether these inconsistencies are attributable to cohort‐specific differences or to the young age groups. For example, BIS is a relatively affluent cohort, with mostly English‐speaking, Anglo/European families.^14^ The mechanisms that explain associations in BIS may be different to those that operate in more heterogeneous contexts. In contrast, BiB is located in a particularly deprived region of the United Kingdom.^15^ Previous research at similarly young ages is sparse. Lower SEP has been associated with higher BP in 2 studies of children aged 5 to 6 years,^11^, ^45^ but not in another multicohort study of those aged 4 to 5 years.^46^ As BP tracks from early childhood across the life course,^47^ further work using repeated measures in the same individuals may identify how early these associations emerge, and whether they are context specific. Some cohorts have begun to collect these data^14^, ^48^; future longitudinal studies should consider more frequent biological measurements during this critical developmental period.

We also observed variation in associations between SEP and cIMT across the cohorts in which it was measured, from early childhood to late adolescence. Boys in lower‐income households in BIS and children in more disadvantaged neighborhoods in LSAC‐CP had higher maximum cIMT compared with their more advantaged counterparts. In ALSPAC, adolescents with mothers with the lowest educational qualifications had lower mean cIMT. Maximum cIMT in BIS and LSAC‐CP refers to the mean of the thickest measurement across several frames of the chosen segment of the carotid artery,^35^ whereas mean cIMT in ALSPAC refers to the mean of measurements across the entire segment.^49^ Some evidence suggests that maximum cIMT is a better predictor of future CVD events than mean cIMT.^50^ Indeed, previous work in LSAC‐CP showed that lower composite SEP was associated with higher maximum, but not mean, cIMT.^35^ These differences in measurement preclude the associations across the cohorts from being directly comparable, particularly at younger ages when interindividual variations may be too small to compare without standardized methods. In adults, maximum cIMT is a stronger predictor of myocardial infarction than mean cIMT.^50^ The extent to which cIMT in adults reflects subclinical atherosclerosis has also been questioned, with the phenotype likely reflecting adaptive processes more than atherosclerotic burden, especially in childhood.^51^, ^52^ Previous work in ALSPAC has shown that cIMT at 17 to 18 years is associated with fat‐free mass and BP, but not metabolic or lifestyle CVD risk factors.^53^ More consistent measurement of cIMT with standardized protocols is needed to identify generalizable associations.^50^

Our study indicates that early‐life socioeconomic exposures at the neighborhood, household, and individual level may be important for CVH in later childhood and adolescence. Each exposure was associated with both vascular and lipid measures from midchildhood. There are likely myriad explanatory mechanisms by which these socioeconomic conditions are reflected in cardiovascular phenotypes. These may include direct pathways, where an exposure results in a direct physiological process, or indirect pathways, where exposures are internalized via neuroendocrine responses.^54^ For example, children in lower‐SEP families may have limited access to a healthy diet or opportunities for physical activity (direct)^55^, ^56^; be exposed to greater air and water pollution (direct)^57^; or experience a stressful family emotional environment because of compromised parental capacity or a reduced sense of belonging attributable to poor neighborhood cohesion (indirect).^58^ We also observed effect modification by sex, particularly for girls. This indicates the potential relevance of sex‐linked and gendered mechanisms that may exacerbate the biological effects of socioeconomic disadvantage in girls.^30^ CIs were wide for models investigating effect modification by ethnicity in BiB. It is plausible that the protective effects of social cohesion and collectivism in the Pakistani community may buffer the adverse effects of low SEP.^59^, ^60^ Our findings highlight the importance of precise structural and social interventions, such as those that improve neighborhood conditions; provide families with the material and social resources needed for their children to thrive; and support mothers with paid parental leave, free childcare, and opportunities for further education.

Mediation analysis indicated that a hypothetical intervention shifting the distribution of BMI in children and adolescents with lower SEP to be the same as that in their more advantaged counterparts would reduce differences in CVH in LSAC‐CP and ALSPAC by between 15% and almost completely. This may have important translational implications at the population level. Obesity is a major CVD risk factor,^13^ and BMI tends to track from childhood to adulthood, with ≈50% of children with obesity also having obesity as adolescents, of whom ≈80% have obesity as adults.^61^, ^62^ Recent systematic reviews have shown that people with persistent overweight from childhood to adulthood have a higher risk of CVD risk factors and clinical outcomes, but not those with overweight‐to‐normal trajectories.^63^, ^64^ These findings underscore the importance of early intervention efforts to alter trajectories of BMI, although BMI‐defined thresholds are not optimal indicators of overweight and obesity.^65^ Body mass and body composition reflect both genetic and environmental factors,^66^ with many of the latter being socially structured. Addressing both upstream mechanisms closely related to SEP and downstream factors, such as diet and physical activity, is likely to be more effective than individual‐level interventions alone.^67^

The main strength of this study is its multicohort design, in which we used prospective data to build evidence for associations between SEP and childhood CVH across various population contexts. We aimed to consider multiple dimensions of SEP across individual, household, and neighborhood levels; similarly, we assessed CVH holistically by multiple vascular and lipid measures. Novel interventional effects methods^32^ allowed us to investigate the potential efficacy of an intervention target, BMI. However, several limitations must be considered. In using cohorts that differ in setting, age group, methods, and sources of bias, it is difficult to disentangle the effects of age and population context, and estimates cannot be directly compared. Analyses should be repeated at older ages in our younger cohorts and in other population‐derived samples. Likewise, differential associations were observed for the cardiovascular measures in each cohort, where some were patterned by SEP in one cohort but not another. Correspondingly, the SEP indicators were associated with a different range of CVH measures in some cohorts compared with others. Without repeated measures, it is difficult to ascertain whether null associations were attributable to age‐related or cohort‐specific contextual and mechanistic factors. We also note difficulties harmonizing household SEP due to data restrictions and highlight that the given measures may not wholly or comparably capture household resources. Although we show evidence that low SEP in early life has implications for CVH in later childhood and adolescence, we also cannot make inferences about the windows of exposure with most biological consequence. Future work may consider using causal mediation analysis to decompose the total effect of early‐life SEP on CVH through later SEP exposure, as well as explore how social mobility and cumulative exposure to SEP may influence outcomes. Unmeasured confounding may also affect study validity and causal inference, given that there are upstream structural, social, and parental factors that could plausibly influence both SEP and child CVH, such as structural racism, parental mental and physical health, and intergenerational factors. In addition, some intermediate confounders (such as diet and physical activity) were either not available or not collected in sufficient participants to be included in mediation analysis, which may affect the validity of some estimates. Furthermore, our findings are only generalizable to similar high‐income contexts. The burden of CVD falls disproportionately on low‐ and middle‐income countries, where it occurs approximately a decade younger on average than in high‐income countries,^1^ but life course mechanisms of risk likely differ considerably.^68^ Finally, self‐reported ethnicity data were limited or unavailable in the study cohorts, despite some being drawn from considerably ethnically diverse populations. Standard practice for the collection of ethnicity data varies across high‐income settings, which may partly account for these limitations.^28^ Where measured, most cohorts were underpowered to explore interactions between ethnicity and SEP. Collecting quality self‐reported ethnicity data remains a priority in longitudinal research and is essential to informing interventions that reduce health inequalities within and among all groups.

## CONCLUSIONS

Our findings suggest that low early‐life SEP may contribute to adverse CVH measures from midchildhood onwards, across cohorts in Australia, Finland, and the United Kingdom. Intervening on BMI and the obesogenic environment may partly reduce socioeconomic differences in vascular and lipid profiles. Future work should consider further disaggregating the pathways by which neighborhood disadvantage, household SEP, and maternal education influence CVH to identify modifiable environmental, social, and behavioral mechanisms. This may inform intervention targets that alter adverse risk trajectories.

## Sources of Funding

K.G. is supported by a University of Melbourne research scholarship. T.M. is supported by a philanthropic fellowship from The DHB Foundation, as managed by equity trustees. D.B. was supported by an Australian National Health and Medical Research Council investigator grant (GTN1175744). K.M. is funded via the UK National Lottery Community Fund (previously the Big Lottery Fund) as part of the A Better Start program (reference 10094849).

## Disclosures

None.

## Supporting information

Data S1‐S5.
Figure S1.
Tables S1‐S32.
References 14,21,35,49,53,69–94

STROBE Statement
