# Supplementary material for: Socioeconomic Position in Early Life and Childhood Cardiovascular Health Measures: An International Cross‐Cohort Study
Source: J Am Heart Assoc. 2026 Aug 27;15(17):e047846. doi: 10.1161/JAHA.125.047846 (PMC13695823; doi:10.1161/JAHA.125.047846)
Supplement: Supplementary file 1 [file JAH3-15-e047846-s001.pdf]

STROBE Statement—checklist of items that should be included in reports of observational studies

	Item No.	Recommendation	Page No.	Relevant text from manuscript
**Title and abstract**	1	(*a*) Indicate the study's design with a commonly used term in the title or the abstract	1	*"cross-cohort"*
		(*b*) Provide in the abstract an informative and balanced summary of what was done and what was found	2	*"We analysed 5 longitudinal cohorts… and lipids (in childhood/adolescence)."*
**Introduction**				
Background/rationale	2	Explain the scientific background and rationale for the investigation being reported	3	*"Cardiovascular disease (CVD) is the… inform timely interventions"*
Objectives	3	State specific objectives, including any prespecified hypotheses	3	"*In this study, we investigated… intervention target."*
**Methods**				
Study design	4	Present key elements of study design early in the paper	3	"*We used data from… data collection."*
Setting	5	Describe the setting, locations, and relevant dates, including periods of recruitment, exposure, follow-up, and data collection	3	"*We used data from… data collection."*
Participants	6	(*a*) *Cohort study*—Give the eligibility criteria, and the sources and methods of selection of participants. Describe methods of follow-up<br>*Case-control study*—Give the eligibility criteria, and the sources and methods of case ascertainment and control selection. Give the rationale for the choice of cases and controls<br>*Cross-sectional study*—Give the eligibility criteria, and the sources and methods of selection of participants	3	"*We used data from… data collection."* [further detail available in supplementary material]
		(*b*) *Cohort study*—For matched studies, give matching criteria and number of exposed and unexposed<br>*Case-control study*—For matched studies, give matching criteria and the number of controls per case		
Variables	7	Clearly define all outcomes, exposures, predictors, potential confounders, and effect modifiers. Give diagnostic criteria, if applicable	4-5	*"Measurement of Early Life Socioeconomic*

				Position…available in Supplemental Methods S4."
Data sources/ measurement	8*	For each variable of interest, give sources of data and details of methods of assessment (measurement). Describe comparability of assessment methods if there is more than one group	4-5	*"Measurement of Early Life Socioeconomic Position…available in Supplemental Methods S4."*
Bias	9	Describe any efforts to address potential sources of bias	5-6	*"We analysed each cohort separately…(intermediate) confounding (Figure 1)."*
Study size	10	Explain how the study size was arrived at	3	*"We included all participants recruited at baseline and who attended a study visit at the chosen wave of data collection."*

Continued on next page

Quantitative variables	11	Explain how quantitative variables were handled in the analyses. If applicable, describe which groupings were chosen and why	4-5	*"Measurement of Early Life Socioeconomic Position…available in Supplemental Methods S4."*
Statistical methods	12	(*a*) Describe all statistical methods, including those used to control for confounding	5-6	*"We conducted data analysis…R.4.1.2."*
		(*b*) Describe any methods used to examine subgroups and interactions	5	*"In secondary analyses…White-British children."*
		(*c*) Explain how missing data were addressed	5	*"Before analysis, we conducted multiple imputation by chained equations…"*
		(*d*) *Cohort study*—If applicable, explain how loss to follow-up was addressed<br>*Case-control study*—If applicable, explain how matching of cases and controls was addressed<br>*Cross-sectional study*—If applicable, describe analytical methods taking account of sampling strategy	3	*"We included all participants recruited at baseline and who attended a study visit at the chosen wave of data collection."*
		(*e*) Describe any sensitivity analyses	5	*"The primary analysis used imputed data; we obtained estimates using complete-case data as a sensitivity analysis."; "In BiB, we additionally conducted sensitivity analysis using a four-category financial security measure as previously used."*
**Results**				
Participants	13*	(a) Report numbers of individuals at each stage of study—eg numbers potentially eligible, examined for eligibility, confirmed eligible, included in the study, completing follow-up, and analysed	N/A	Provided in supplementary material
		(b) Give reasons for non-participation at each stage	N/A	Provided in supplementary material
		© Consider use of a flow diagram	N/A	
Descriptive data	14*	(a) Give characteristics of study participants (eg demographic, clinical, social) and information on exposures and potential confounders	6	*"This study included…boys [43.7%]) (Table 1)."*
		(b) Indicate number of participants with missing data for each variable of interest	6	Table 1
		(c) *Cohort study*—Summarise follow-up time (eg, average and total amount)	N/A	Provided in supplementary material

Outcome data	15*	*Cohort study*—Report numbers of outcome events or summary measures over time	6	Table 1
		Case-control study—Report numbers in each exposure category, or summary measures of exposure		
		Cross-sectional study—Report numbers of outcome events or summary measures		
Main results	16	(*a*) Give unadjusted estimates and, if applicable, confounder-adjusted estimates and their precision (eg, 95% confidence interval). Make clear which confounders were adjusted for and why they were included	6-9	*"Neighborhood Disadvantage and Cardiovascular Health… White-British children (Tables S25, S26, and S27)."*
		(*b*) Report category boundaries when continuous variables were categorized	7-9	Figures 1-3
		(*c*) If relevant, consider translating estimates of relative risk into absolute risk for a meaningful time period	7-9	e.g., *"20% reduction in PWV differences (interventional indirect effect [IIE] = 0.02 m/s; 95% CI, 0.0-0.04; P=0.008)"*

Continued on next page

Other analyses	17	Report other analyses done—eg analyses of subgroups and interactions, and sensitivity analyses	6-9	e.g., *"Subgroup analysis showed that girls in the most disadvantaged neighborhoods in NFBC1986 may have higher triglycerides (β = 0.1 mmol/L; 95% CI, -0.0-0.1; P=0.07)…"*
**Discussion**				
Key results	18	Summarise key results with reference to study objectives	9	*"This multicohort study of 21,500 participants from five longitudinal birth cohorts aged 4-18 years suggests…"*
Limitations	19	Discuss limitations of the study, taking into account sources of potential bias or imprecision. Discuss both direction and magnitude of any potential bias	11	*"However, several limitations must be considered…"*
Interpretation	20	Give a cautious overall interpretation of results considering objectives, limitations, multiplicity of analyses, results from similar studies, and other relevant evidence	12	*"Our findings suggest that low early-life SEP may contribute to adverse CVH measures from mid-childhood onwards, across cohorts in Australia, Finland, and the UK. Intervening…"*
Generalisability	21	Discuss the generalisability (external validity) of the study results	11	*"Our findings are only generalizable to similar high-income contexts…"*
**Other information**				
Funding	22	Give the source of funding and the role of the funders for the present study and, if applicable, for the original study on which the present article is based	12	Under: *"Funding Sources"*

*Give information separately for cases and controls in case-control studies and, if applicable, for exposed and unexposed groups in cohort and cross-sectional studies.

Note: An Explanation and Elaboration article discusses each checklist item and gives methodological background and published examples of transparent reporting. The STROBE checklist is best used in conjunction with this article (freely available on the Web sites of PLoS Medicine at http://www.plosmedicine.org/, Annals of Internal Medicine at http://www.annals.org/, and Epidemiology at http://www.epidem.com/). Information on the STROBE Initiative is available at www.strobe-statement.org.
